# Cytotoxicity of a Lipid-Rich Extract from Native Mexican Avocado Seed (*Persea americana* var. drymifolia) on Canine Osteosarcoma D-17 Cells and Synergistic Activity with Cytostatic Drugs

**DOI:** 10.3390/molecules26144178

**Published:** 2021-07-09

**Authors:** Salvador Padilla-Arellanes, Rafael Salgado-Garciglia, Marisol Báez-Magaña, Alejandra Ochoa-Zarzosa, Joel Edmundo López-Meza

**Affiliations:** 1 Centro Multidisciplinario de Estudios en Biotecnología-FMVZ, Universidad Michoacana de San Nicolás de Hidalgo, Posta Veterinaria, Morelia 58893, Mexico; salvador.padilla@umich.mx (S.P.-A.); solbaez2@gmail.com (M.B.-M.); ochoaz@umich.mx (A.O.-Z.); 2 Instituto de Investigaciones Químico-Biológicas, Universidad Michoacana de San Nicolás de Hidalgo, Morelia 58030, Mexico; rafael.salgado@umich.mx

**Keywords:** *Persea americana*, osteosarcoma, apoptosis, avocado, canine, synergism

## Abstract

Osteosarcoma is the most common malignant bone tumor in both children and dogs. It is an aggressive and metastatic cancer with a poor prognosis for long-term survival. The search for new anti-cancer drugs with fewer side effects has become an essential goal for cancer chemotherapy; in this sense, the bioactive compounds from avocado have proved their efficacy as cytotoxic molecules. The objective of this study was to determine the cytotoxic and antiproliferative effect of a lipid-rich extract (LEAS) from Mexican native avocado seed (*Persea americana* var. drymifolia) on canine osteosarcoma D-17 cell line. Also, the combined activity with cytostatic drugs was evaluated. LEAS was cytotoxic to D-17 cells in a concentration-dependent manner with an IC_50_ = 15.5 µg/mL. Besides, LEAS induced caspase-dependent cell apoptosis by the extrinsic and intrinsic pathways. Moreover, LEAS induced a significant loss of mitochondrial membrane potential and increased superoxide anion production and mitochondrial ROS. Also, LEAS induced the arrest of the cell cycle in the G0/G1 phase. Finally, LEAS improved the cytotoxic activity of cisplatin, carboplatin, and in less extension, doxorubicin against the canine osteosarcoma cell line through a synergistic effect. In conclusion, avocado could be a potential source of bioactive molecules in the searching treatments for osteosarcoma.

## 1. Introduction

Cancer represents a serious human health worldwide problem; recently data of GLOBOCAN indicate that the number of new cases in 2020 has risen to 19.3 million. Besides, cancer is the leading cause of disease-associated death in dogs and it is estimated that 50% of dogs >10 years old develop some type of cancer [1,2,3]. Osteosarcoma (OSA) is the most common malignant bone tumor in both humans and dogs, although the incidence in dogs is ten times higher [4,5]. OSA is more common in larger and giant breeds of dogs, such as the Rottweiler and Great Pyrenees, among others [6,7]. OSA is an aggressive and highly metastatic cancer, with significant morbidity and a poor prognosis for long-term survival and occurs spontaneously in diverse skeletal locations, such as the appendicular skeleton, axial skeleton, and extraskeletal sites [8]. In dogs, despite appropriate surgical and chemotherapeutic protocols only approximately 50% of affected dogs survive the first year, and fewer (~20%) survive more than two years after diagnosis [9]. Also, research in both canine and human OSA has demonstrated that both species share similar genetic abnormalities [8]. For the above mentioned, and given the high frequency of OSA in canines, the dog is considered an attractive model to study and learn about the origin of this cancer, as well as to evaluate new drugs or treatments that may have potential use in humans too [3,10,11].

The OSA treatment includes conventional approaches such as chemotherapy. Diverse studies have demonstrated the efficiency of cytostatic drugs such as carboplatin, cisplatin, and doxorubicin; however, these still have a low therapeutic index and severe side effects [12]. One strategy to solve these problems is the use of combination therapy, which may reduce the negative side effects. These treatment limitations have led to the search for new cytotoxic compounds which are more selective and aggressive towards cancer cells. In this sense, plant constituents have beneficial effects in the prevention and treatment of cancer, which are attributable essentially to the phytochemicals or secondary metabolites [13]. Recently, the cytotoxicity and the antiproliferative effects of pure artemisinin and a hydroalcoholic extract obtained from *Artemisia annua* on the D-17 canine osteosarcoma cell line were reported [14]. Also, the effects of two extracts (turmeric root and rosemary leaf extract) on three different canine cell lines (C2 mastocytoma, and CMT-12 mammary carcinoma, D-17 osteosarcoma cell line) were assessed [15]. These reports highlight the potential of plant extracts in the search for new alternatives for OSA treatment.

The avocado tree (*Persea americana* Mill.) has been used to treat various health conditions in humans [16]. In particular, avocado anti-cancer activities have been associated with more than 20 groups of bioactive compounds present in the tree and the fruit [17,18], highlighting the long-chain lipid molecules contained in the fruit, such as the long-chain fatty acids and their derivatives (avocatins, pahuatins, persenins, and the polyhydroxylated fatty alcohols) [18,19,20,21,22]. The native Mexican avocado fruit (*P. americana* var. drymifolia) represents a source of bioactive molecules with anti-cancer and anti-inflammatory properties [23]. Recently, we reported that a lipid-rich extract from native Mexican avocado seed (LEAS, 1–200 ng/mL) reduces *Staphylococcus aureus* internalization into bovine mammary epithelial cells and regulates innate immune response [24]. Also, LEAS has shown cytotoxic activity on colorectal cancer Caco-2 cells (IC_50_ = 28 µg/mL) inducing apoptosis. Besides, LEAS inhibited fatty acid oxidation and increased the superoxide production and mitochondrial ROS; also, it stimulated secretion of cytokines IL-6, IL-8, and IL-10; whereas IL-1β secretion was inhibited (~50%) [22]. However, the effect of LEAS on OSA is unknown. For the above mentioned, the present study aimed to evaluate the cytotoxic and antiproliferative effects of LEAS (*P. americana* var. drymifolia) on canine osteosarcoma cell line D-17. Also, the combined activity of LEAS with cytostatic drugs was analyzed.

## 2. Results

### 2.1. LEAS Is Cytotoxic for D-17 Cells

**Figure 1:**
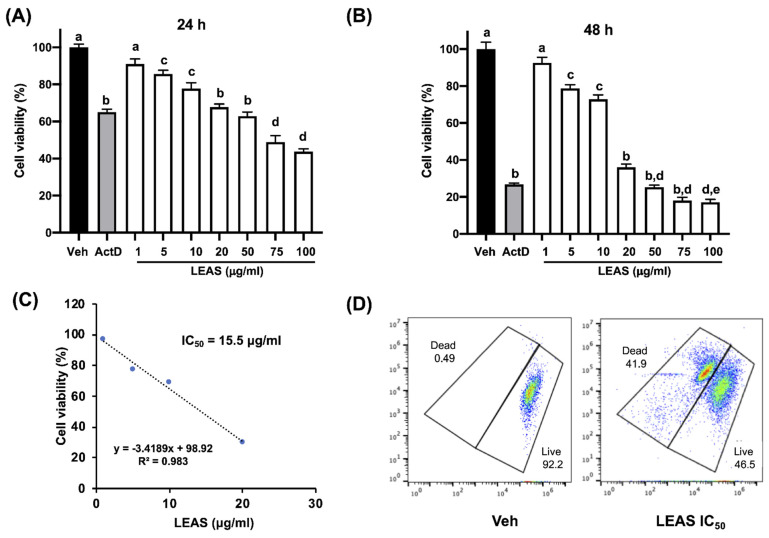
Effect of LEAS on canine osteosarcoma D-17 cell line viability. Cells were treated with LEAS (1, 5, 10, 20, 50, 75, and 100 μg/mL) and cell viability was evaluated by MTT assays at 24 h (**A**) and 48 h (**B**). Cell viability is shown with respect to cells treated with vehicle (DMSO 0.1%). Actinomycin D (ActD) was used as a positive control (80 μg/mL). Data represent the mean of three independent experiments performed in triplicate. Different letters denote significant differences within the treatments (one-way ANOVA and Tukey’s pairwise comparison, *p* < 0.05). (**C**) Linear regression analysis of the concentration-response to calculate the half-maximal inhibitory concentration (IC_50_) of LEAS on the D-17 cell line at 48 h. (**D**) In vitro assessment of LEAS IC_50_ (15.5 μg/mL) by flow cytometry at 48 h using SYTO^TM^ 9 green-fluorescent nucleic acid stain and propidium iodide. Representative plots of different treatments are shown.

The canine OSA D-17 cell line was treated with increasing concentrations of LEAS (1 to 100 μg/mL) for 24 and 48 h, and effects on cell viability were assessed. LEAS showed concentration-dependent cytotoxic effects on D-17 cells. The results showed that after 24 h of incubation, D-17 cell viability decreased when cells were treated with LEAS 20 µg/mL (29%) (Figure 1A). However, cytotoxicity was increased after 48 h at 10 µg/mL (31%) and 20 µg/mL (70%) (Figure 1B). The half-maximal inhibitory concentration at 48 h was calculated as IC_50_ = 15.5 µg/mL, which was corroborated by flow cytometry (Figure 1C,D); this concentration was used in the rest of the experiments. Also, LEAS did not exhibit hemolytic action at any concentration used, suggesting that these molecules do not have a cytotoxic effect on normal blood cells. On the other hand, LEAS was significantly less cytotoxic on MDCK cells with an IC_50_ = 50 µg/mL, which was three times higher than that showed on the osteosarcoma cell line (Figure S1). According to these results, further experiments were performed with the LEAS IC_50_ on D-17 cells to establish the dead mechanism activated.

### 2.2. LEAS Does Not Affect the Cell Membrane of D-17 Cells

**Figure 2:**
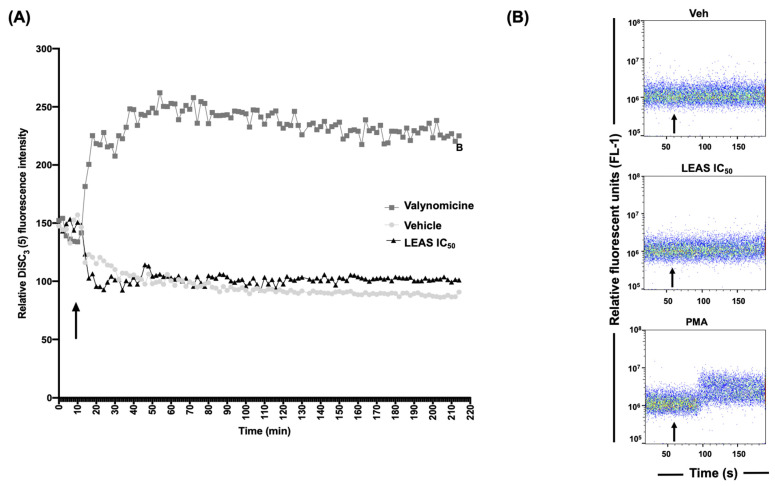
LEAS does not affect the cytoplasmic membrane integrity in D-17 cells. (**A**) Changes in the cytoplasmic membrane potential of D-17 cells were evaluated using membrane potential-sensitive dye. Cells were previously incubated with 200 μM of the dye DiSC3(5) for 30 min at 37 °C and then treated with LEAS IC_50_ (15.5 µg/mL). Valinomycin (0.2 mM) was used as a positive control. (**B**) The efflux of cytosolic calcium was analyzed by flow cytometry. Measurements were assayed by 3 min, and at minute one the cells were treated with LEAS IC_50_ (15.5 µg/mL), vehicle, or PMA 3 μM (acetate phorbol myristate) as a positive control. Representative plots are shown. Arrows indicate the time at which the treatments were added. Vehicle = DMSO (0.1%).

We evaluate the calcium efflux and membrane electrical potential to determine if LEAS cytotoxicity on D-17 cells was associated with cell membrane damage. The results indicated that cytotoxicity induced by LEAS was not a result of cell membrane compromise as both calcium efflux and membrane electrical potential were not altered (Figure 2).

### 2.3. LEAS Cytotoxicity on D-17 Cells Is Related to Apoptosis Induction

**Figure 3:**
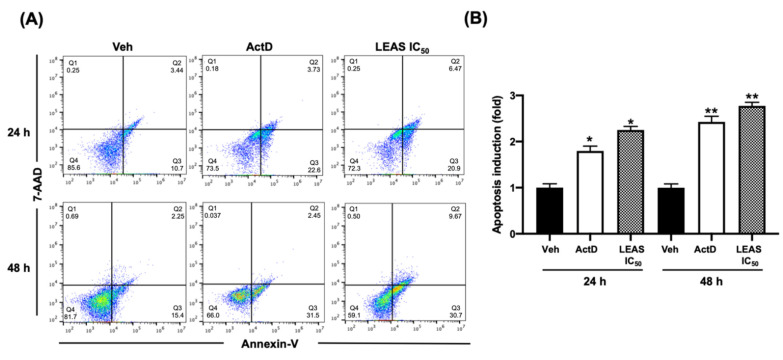
Apoptosis induced by LEAS in D-17 cells. (**A**) Cells treated with LEAS IC_50_ (15.5 µg/mL) for 24 h and 48 h were analyzed by flow cytometry using Annexin-V and /AAD staining to establish the apoptotic rate. Cells sorted into quadrants Q1, Q2, Q3 and, Q4 represent necrotic, late apoptotic, early apoptotic, and viable (live) populations, respectively. Representative plots are shown. (**B**) The graphic shows the apoptosis fold-change in the treatments at times evaluated. Each bar shows the mean of triplicates ± SE. A minimum of 10,000 events per sample was collected. * and ** indicate statistically significant differences with respect to vehicles (*p* < 0.05) at the different times evaluated. ActD = Actinomycin D (80 μg/mL). Veh = vehicle (DMSO 0.1%).

**Figure 4:**
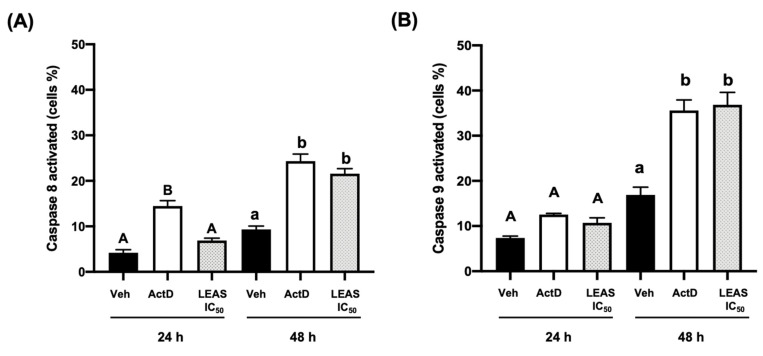
Caspase-8 and caspase-9 activation by LEAS in D-17 cells. Cells treated with LEAS IC_50_ (15.5 μg/mL) for 24 h and 48 h were analyzed by flow cytometry to caspase-8 (**A**) and caspase-9 activation (**B**). Representative plots are shown. The graphic shows percentage of cells with caspase-activated for each time of treatment. Each bar shows the mean of triplicates ± SE of two independent experiments. Different letters denote significant differences within the treatments at the time evaluated (one-way ANOVA and Tukey’s pairwise comparison, *p* < 0.05). ActD = Actinomycin D (80 μg/mL). Veh = vehicle (DMSO 0.1%).

To evaluate the death mechanism activated by LEAS on D-17 cells, the apoptosis rate was assessed by flow cytometry. Cells were treated with the LEAS IC_50_ (15.5 µg/mL) for 24 and 48 h, and apoptosis was evaluated (Figure 3A). LEAS IC_50_ induced apoptosis in D-17 cells in a time-dependent manner, and the apoptosis rate was similar (>37%) to that exhibited by actinomycin D at 48 h (Figure 3B). Also, the results showed that necrosis did not increase significantly in cells treated with LEAS. Besides, the activity of caspase-8 and caspase-9 was measured, which are characteristic of the extrinsic and intrinsic pathways, respectively. At 24 h of treatment, the activation of both caspases was not increased significantly. However, at 48 h the activation of both caspases was detected (caspase-8, 22% and caspase-9, 37%); indicating that both pathways were activated (Figure 4).

### 2.4. LEAS Cytotoxicity Is Associated with Loss of Mitochondrial Membrane Potential and ROS Production in D-17 Cells

**Figure 5:**
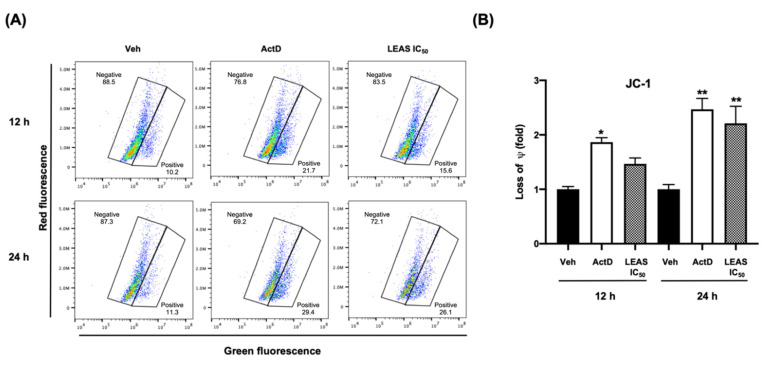
LEAS generates the loss of mitochondrial membrane potential (ΔΨm) in D-17 cells. (**A**) The mitochondrial membrane potential was evaluated by flow cytometry using the JC-1 dye. Cells were treated for 12 h and 24 h with LEAS IC_50_ (15.5 μg/mL), vehicle, or Act D. Representative plots are shown. (**B**) The graphic shows fold-change values for each time of treatment. Each bar shows the mean of triplicates ± SE of two independent experiments. “*” and “**” Indicates statistically significant differences with relation to the vehicle at the time evaluated (*p* < 0.05). ActD = Actinomycin D (80 μg/mL). Veh = vehicle (DMSO 0.1%).

**Figure 6:**
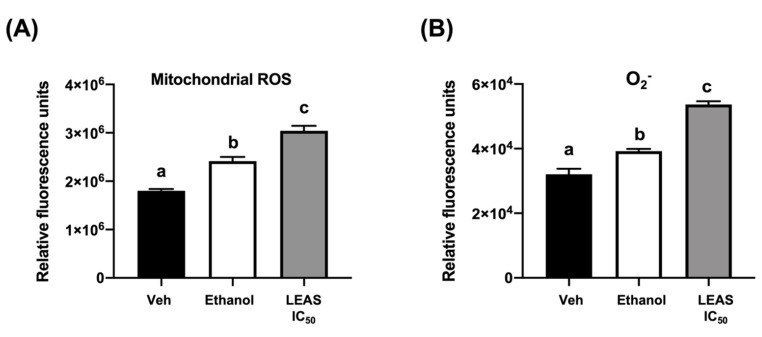
LEAS increased the production of ROS in D-17 cells. The production of mitochondrial ROS (**A**) and superoxide anion (**B**) were assessed by flow cytometry using the dyes DHE (5 μM) and DHR (10 μM), respectively. Cells were treated for 48 h with LEAS IC_50_ (15.5 μg/mL), vehicle (DMSO 0.1%), or ethanol (12%) as a positive control. The graphic shows fold-change values for each treatment. Each bar shows the mean of triplicates ± SE of two independent experiments. Different letters indicate statistically significant differences in relation to the vehicle (*p* < 0.05).

Mitochondrial membrane potential was assessed using the dye JC-1 by flow cytometry. Under normal conditions, the electrochemical gradient of intact mitochondria results in aggregation of the dye, which fluoresces red. In contrast, fluorescent dye escapes from the depolarized mitochondria of apoptotic cells, and the non-aggregated dye fluoresces green. We demonstrated that LEAS caused a loss of mitochondrial membrane potential in D-17 cells treated with LEAS at 12 and 24 h (Figure 5), increasing cells fluorescing green (>39%) when compared with cells treated only with vehicle (<18%) (Figure 5B). Because the induction of apoptosis is usually correlated with the activity of ROS disrupting mitochondrial membrane potential, we measured the generation of O_2_^−^ and ROS by flow cytometry. We showed that the addition of LEAS treatment significantly increased the production of O_2_^−^ (65%) and ROS (72%) in D-17 cells compared with vehicle (Figure 6). Taken together, these results indicated that LEAS induced production of O_2_^−^ and ROS and, consequently, loss of mitochondrial membrane potential favoring apoptosis by intrinsic pathway.

### 2.5. LEAS Induces Cell Cycle Arrest in D-17 Cells

**Figure 7:**
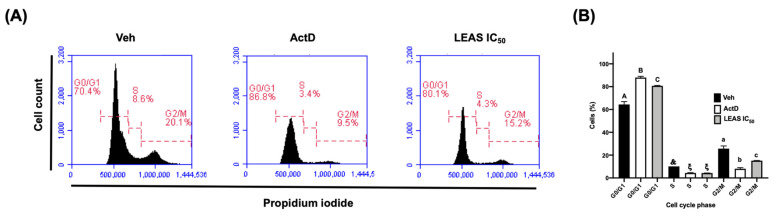
Effect of LEAS on cell cycle progression. (**A**) The cell cycle phases were determined by flow cytometry using propidium iodide. Cells were treated for 48 h with LEAS IC_50_ (15.5 μg/mL), vehicle (DMSO 0.1%), or ActD (actinomycin D, 80 μg/mL). (**B**) The graphic shows the percentage of cells in the different cell cycle phases. Each bar shows the mean of triplicates ± SE. For each sample, 20,000-gated events were acquired. Statistically significant differences of each phase of the cell cycle between the treatments are indicated (*p* < 0.05). ActD = Actinomycin D (80 μg/mL). Veh = vehicle (DMSO 0.1%).

To investigate the effect of LEAS treatment on cell cycle progression in D-17 cells, these were incubated with IC_50_, and then flow cytometry was used to assay the DNA content per cell. LEAS increased significantly the proportion of cells in G0/G1 phase at 48 h (82%) with relation to control cells (66%), which was correlated with a decrease in the proportion of cells in G2/M phase at 48 h (10%) with relation to control (19%) (Figure 7).

### 2.6. LEAS Shows a Synergistic Effect with Cytotoxic Drugs in D-17 Cells

**Figure 8:**
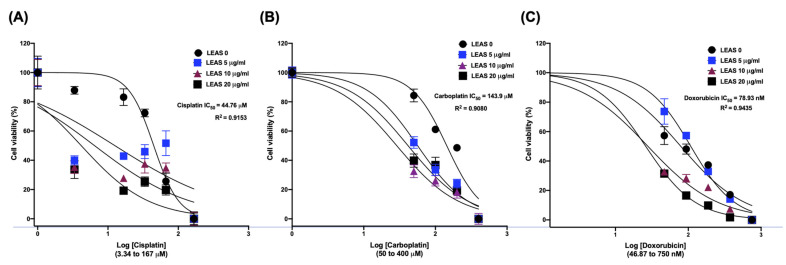
Synergistic effect of LEAS with cytostatic drugs on cell viability of D-17 cells. (**A**) Cisplatin, (**B**) Carboplatin, and (**C**) Doxorubicin. D-17 cells were subjected to the simultaneous treatment with LEAS (5, 10, 20, 50 µg/mL), cisplatin (3.34, 16.7, 33.4, 66.8 μM), carboplatin (50, 100, 200, 400 μM) or doxorubicin (46.8, 97.7, 187, 350 nM) and incubated for 72 h. The viable cells were determined by MTT assay and the IC_50_ was estimated. Controls consisting of cells treated with the individual compounds were included. All treatments were evaluated in two independent experiments performed by triplicate.

Lastly, we evaluated the cytotoxic effect of LEAS in combination with cytostatic anticancer drugs cisplatin, carboplatin, and doxorubicin on osteosarcoma D-17 cells. Cell viability analysis showed an IC_50_ = 44.76 µM for cisplatin (Figure 8A); notably, the cytotoxicity was increased by LEAS through synergistic action, highlighting the combination of LEAS (5 µg/mL) and cisplatin (3.34 µM) which decreased cell viability by 60% when compared to vehicle-treated cells. Likewise, LEAS improved the carboplatin cytotoxicity by synergistic action. Carboplatin showed an IC_50_ = 143.9 µM on osteosarcoma cells (Figure 8B); however, in combination with LEAS, we observed a significant decrease in cell viability, even the lowest concentration of both showed a synergistic effect with a reduction in cell viability (48%). Finally, only three doxorubicin combinations (Figure 8C) showed a synergistic effect (LEAS 10 µg/mL and doxorubicin 375 nM; LEAS 20 µg/mL and doxorubicin 93.75 or 187.5 nM). These results indicate that LEAS has a synergistic effect with cisplatin, carboplatin, and to a lesser extent with doxorubicin, improving its cytotoxicity on D-17 cells.

## 3. Discussion

Avocado fruit is rich in lipids and diverse studies have shown their anticancer activities, which vary with the composition and the chemical nature of the molecules [20,21]. The lipid proportions vary among the varieties of avocado, but we focused on the Mexican native variety (*P. americana* var. drymifolia) which shows the higher oil content (20–30%) [25]. The pulp lipids of the native Mexican avocado fruit have attracted attention due to their properties; however, the nutraceutical properties of seed lipids have been poorly studied. In this study, the cytotoxic effects of a lipid-rich extract from avocado Mexican native seeds (LEAS) on the D-17 osteosarcoma cell line were evaluated. LEAS decreased D-17 cell viability through apoptosis induction, which was related to ROS production and depolarization of the mitochondrial membrane. Also, LEAS favors the arrest of the cell cycle in the G0/G1 phase. Besides, the LEAS improved the cytotoxicity of different cytostatic drugs and could have potential use in combined treatment for OSA control.

The side effects and toxicity of chemotherapy in patients with cancer remain a major problem; therefore, the search for safer alternatives such as natural products to be used as mono or adjunct therapy with the standard drugs is a priority in anti-cancer research [10,13]. Numerous studies have evaluated the cytotoxic properties of avocado compounds against different types of cell lines from leukemia, colon, oral, and prostate cancer [17,18,22,26]. However, to our knowledge, avocado lipids have not been evaluated on bone tumors, such as osteosarcomas, which are the most prevalent bone neoplasm in humans and dogs. In this work, LEAS exhibited cytotoxic effects on D-17 canine osteosarcoma cells (IC_50_ = 15.5 μg/mL) in a concentration-dependent manner (Figure 1). Similar results to LEAS on D-17 cells have been reported with other plant extracts. Isani et al. [14] reported the cytotoxic effects of an extract from sweet absinthe and artemisinin with an IC_50_ of 65 μM for the hydroalcoholic extract and 548 μM for the pure standard. Also, the cytotoxicity of baicalein (a flavonoid) was evaluated on three osteosarcoma cells (HMPOS, D-17, and OS 2.4) with cytotoxic concentrations from 1 to 25 μM [27]. Concerning the cytotoxic activity of avocado seed extracts on cancer cells, Alkhalf et al. [28] evaluated a lipid seed extract that exhibited anti-cancer activities in a concentration-dependent manner on the HCT116 (colon cancer) and the HePG2 (liver cancer) human cell lines. Likewise, chloroform extract from avocado seed showed strong cytotoxic activity against the MCF-7 cell line with an IC_50_ = 94.87 μg/mL [29]. On the other hand, some non-steroidal anti-inflammatory drugs, such as carprofen and tolfenamic acid were cytotoxic for D-17 cells [30]. Earlier, we reported the anti-inflammatory effects of LEAS on mammary epithelial cells [24]. In further studies it will be necessary to evaluate this activity on D-17 cells and to determine its contribution to the LEAS cytotoxic activity. Unlike other cancers, the availability of commercial canine osteosarcoma cell lines is not wide enough. However, due to the interest that canine osteosarcoma represents, both for the health of dogs as a model for human osteosarcoma, new cell lines are being established; this will open the possibility that in the future the effects of LEAS could be evaluated in other lines from canine osteosarcoma. Besides, recently we reported that LEAS is cytotoxic towards human colon Caco-2 cells with an IC_50_ of 28 μg/mL [22], and recent results from our laboratory showed that LEAS is cytotoxic towards MCF-7 human breast cancer cells and the mouse melanoma cell line B16-F0. These results show that LEAS is cytotoxic towards cell lines of different origins and with other genetic backgrounds. On the other hand, LEAS was significantly less cytotoxic on MDCK cells with an IC_50_ = 50 μg/mL, which was three times higher than LEAS cytotoxicity on D-17 cells (Figure S1), indicating the selective toxicity of LEAS on the osteosarcoma cells.

In most cases, cancer cells maintain accelerated growth and longer survival than normal cells by suppressing apoptosis. In this work, the LEAS cytotoxicity on D-17 cells was related to apoptosis induction through both extrinsic and intrinsic pathways activation. The decrease in cell viability by LEAS was correlated with an increase in apoptotic activity, because the percentage of apoptotic cells was significantly higher in LEAS treated cells compared to control cells; besides, an increase in the activation of caspase 8 and caspase 9 was detected (Figure 3 and Figure 4). In agreement, other groups have demonstrated that avocado lipids activate both extrinsic and intrinsic apoptosis simultaneously in oral [20] and breast cancer cells [19]. In agreement with the apoptosis induction, LEAS (IC_50_ 15.5 μg/mL) induced the loss of mitochondrial membrane potential of D-17 cells (Figure 5). A similar effect has been reported for avocado fatty acid derivatives, which induced the loss of mitochondrial potential in oral cancer cells [20]. Dysfunction of mitochondrial membrane is an important mechanistic feature of apoptosis of cancer cells treated with different drugs. The opening of mitochondrial permeability transition pores leads to depolarization of transmembrane potential, favoring the release of cytochrome c and pro-apoptotic proteins. Also, LEAS induced ROS production, which is consistent with the activation of the intrinsic pathway. Similar results were obtained in a study treating D-17 cells with the flavonoid myricetin [31]. In another study using dihydroartemisinin, cell death via oxidative damage was induced by ROS production in osteosarcoma cells [32].

The growth of tumor cells may be inhibited at any phase of the cell cycle, which results in cell cycle arrest. Phytochemicals extracted from *P. americana* induce cell cycle arrest [33]. Our data indicate that LEAS affects the cell cycle of D-17 cells, arresting the cells in G0/G1 phase. In agreement, betulinic acid (a triterpenoid) induced the cell cycle arrest of the D-17 cell line in the S phase [34]. Also, the flavonoid baicalein arrested the cell cycle of three canine osteosarcoma cell lines with a significant increase in G0/G1 and a decrease in G2/M [27].

Cancer treatment with cytostatic drugs has been used in the last 25 years, but significant adverse effects are very common. For this reason, new drugs and therapeutic targets are needed to improve the outcome of dogs suffering OSA and to reduce the long-term toxicities associated with the standard treatments [10,35]. The combination of two or more therapeutic agents permits diminished drug dosages and consequently toxicity, and also reduces the development of resistance by target cells and provides the potential for synergism of drug effects. The most common chemotherapeutic agents used for OSA treatment in dogs are doxorubicin and platinum-based compounds such as carboplatin and cisplatin [10]. In this work, the cytotoxicity of doxorubicin, carboplatin, and cisplatin was evaluated in combination with LEAS on D-17 cells (Figure 8). Our drug combination studies demonstrated synergism of the three compounds with LEAS and an enhancement in the sensitivity of D-17 cells to these cytostatic drugs was observed. The synergism of LEAS with the drugs could limit some of the acute and long-term toxicities associated with high doses.

## 4. Materials and Methods

### 4.1. Reagents, Cell Lines, and Cell Culture

The osteosarcoma D-17 (ATCC^®^ CCL-183™) cell line was purchased from Cientifica Senna (Ciudad de México, México) and MDCK (ATCC^®^ CCL-34™) cell line was kindly donated by Laura Cobos-Marín (UNAM, México). Both cell lines were routinely cultured according to the supplier’s recommendations. Cells were grown in Dulbecco’s modified Eagle’s medium/nutrient F-12 Ham (DMEM/F12K, Sigma-Aldrich, St. Louis, MO, USA) supplemented with 10% fetal calf serum (Equitech Bio, Kerrville, TX, USA), 100 U/mL penicillin/streptomycin (Gibco), and 1 µg/mL amphotericin B (Invitrogen) at 37 °C in a humidified atmosphere with 5% CO_2_. All experiments were performed using cell lines maintained at low passage numbers (5–10 passages). Cisplatin, carboplatin, and doxorubicin were purchased from Sigma and work solutions were prepared in Dulbecco’s modified Eagle’s medium/nutrient F-12 Ham.

### 4.2. Lipid-Rich Extract from Avocado Seed (LEAS)

Mexican avocado fruits (*P. americana* var. drymifolia) were collected when physiological maturity was achieved in Michoacán, Mexico. The LEAS was obtained as reported [24]. Briefly, frozen avocado seeds were crushed, and the powder was extracted in a Soxhlet apparatus for 14 h with hexane (C_6_H_14_, J.T. Baker). This fraction was filtered (Whatman filter, Sigma, St. Louis, MO, USA) and cooled for 12 h at −18 °C to obtain the LEAS crystals, which were recovered by discarding the supernatant and then drying with gas nitrogen. This extract contains abundant molecules of 17–21 carbon aliphatic chains with hydroxyl groups such as aliphatic acetogenins and long-chain fatty acids. The extract characterization was previously reported [24]. The crystals were resuspended in 5% DMSO (dimethylsulfoxide, Sigma, St. Louis, MO, USA). The evaluated concentrations of LEAS were: 1, 5, 10, 20, 50, 75, 100, and 150 µg/mL, and the final concentration of vehicle for all the experiments was DMSO 0.1%, which was used as control.

### 4.3. MTT Viability Assay

Cell viability was assessed by 3-(4,5-dimethylthiazol-2-yl)-2,5-diphenyltetrazolium bromide (MTT; Sigma, St. Louis, Missouri, MO, USA) assays after 24 and 48 h of treatment with LEAS, and the half-maximal inhibitory concentration (IC_50_) was calculated as reported [22]. For this, D-17 and MDCK cell lines were plated at a density of 10,000 cells per well in 96-well tissue culture-treated plates (Corning Inc., Corning, New York, NY, USA) and synchronized by starvation using a medium without supplements for 24 h. Cells were treated with vehicle (DMSO), actinomycin D (80 µg/mL, Sigma), or LEAS (ranging from 1 to 100 µg/mL). Briefly, MTT assays were performed by adding 10 μL of MTT dye (5 mg/mL) in phosphate-buffered saline (PBS) to each well and incubated for 4 h at 37 °C in 5% CO_2_. Finally, formazan crystals were solubilized in acid isopropanol (100 μL, 95% isopropanol, and 5% of 1 N HCl). The absorbance measurements were carried out in a spectrophotometric plate reader (Bio Rad) at a wavelength of 595 nm. Cell viability results are reported as the percentage of viable cells with respect to cells treated with the vehicle. The IC_50_ was calculated by regression analysis using Excel (Microsoft) and corroborated by flow cytometry with SYTO*9 green-fluorescent nucleic acid stain and propidium iodide in a BD Accuri^TM^ C6 flow cytometer (BD Biosciences, San Jose, CA, USA) according to the manufacturer´s instructions. For the rest of the experiments on the D-17 cells, the LEAS IC_50_ calculated was used.

### 4.4. Calcium Efflux Testing

Calcium efflux was assessed by flow cytometry in a BD Accuri^TM^ C6 flow cytometer using a calcium assay kit (BD Biosciences, San Jose, CA, USA) according to the manufacturer’s instructions. Briefly, D-17 cells (1 × 10^5^/mL) were cultured and synchronized in 100 mm flasks, then incubated with the indicator dye for 1 h at 37 °C. The baseline fluorescence was established (1 min), and then the treatments were added (LEAS IC_50_ or vehicle). The measurements were performed for 3 min without interrupting the data collection. The changes of the fluorescence intensity in cell populations in response to the treatments were monitored using flow cytometry. Phorbol myristate acetate (3 mM; PMA, Sigma) was used as a positive control. Data were analyzed using FlowJo Software v. 10.4.2 (TreeStar Inc., Ashland, OR, USA).

### 4.5. Measurement of the Membrane Potential

The changes in membrane potential of D-17 cells were measured using the DiSC3(5) dye (3,3´-dipropylthiadicarbocyanine iodide, Sigma) as described [36]. Briefly, D-17 cells (1 × 10^4^/mL) were seeded in 96-well black-wall plates and cultured for 24 h. Then, they were washed two times with Hank’s-HEPES buffer and incubated with DiSC3(5) 0.2 mM (dissolved in Hank’s-HEPES buffer) for 30 min in a CO_2_ incubator. LEAS IC_50_ and vehicle were added to each well, and subsequent changes in the fluorescence intensity were monitored for 2 h in a Varioskan spectrophotometer (Thermo Scientific, Waltham, MA, USA). Valynomicin at 0.2 mM (Sigma) was used as a positive control.

### 4.6. Assessment of Mitochondrial Membrane Potential (ΔΨm)

The effect of LEAS on the ΔΨm of D-17 cells was evaluated by flow cytometry using JC-1 dye (BD Biosciences, San Jose, CA, USA) that allows differentiating healthy cells (red fluorescence) from those with mitochondrial damage (green fluorescence). For this, D-17 cells (4 × 10^5^/mL) were cultured in 24-well plates and treated with LEAS IC_50_ or vehicle and stained with JC-1 dye for 15 min at 37 °C in the dark, according to the manufacturer’s instructions. The cells were subsequently washed twice with assay buffer, and their fluorescence was measured in a BD Accuri^TM^ C6 flow cytometer (BD Biosciences, San Jose, CA, USA). Data were analyzed using the FlowJo Software v. 10.4.2 (TreeStar Inc., Ashland, OR, USA).

### 4.7. Determination of Reactive Oxygen Species (ROS)

Superoxide anion (O_2_^−^) and mitochondrial ROS production were measured by flow cytometry in a BD Accuri^TM^ C6 flow cytometer (BD Biosciences, San Jose, CA, USA) using dihydroethidium (DHE, 5 µM, molecular probes) and dihydrorhodamine-123 (DHR, 15 µM, molecular probes), respectively. D-17 cells (4 × 10^5^/mL) were cultured and synchronized for 24 h. Thereafter, they were incubated for 48 h with LEAS IC_50_ or vehicle. Next, cells were recovered by trypsinization, washed with PBS, and incubated with DHR or DHE for 2 h in a 5% CO_2_ atmosphere at 37 °C in darkness. Finally, cells were washed and analyzed. Ethanol (12% *v/v*) was used as a positive control. A total of 10,000 events were evaluated.

### 4.8. Apoptosis Analysis

Flow cytometry was used to detect the binding of annexin V to phosphatidylserine on the surface of cells. Briefly, D-17 cells were plated in 24-well plates and synchronized for 24 h. Thereafter, the cells were treated with LEAS for 24 and 48 h before being harvested by trypsinization and centrifugation. Cells were washed with PBS and resuspended in 100 μL binding buffer according to the manufacturer’s instructions (BD Biosciences, San Jose, CA, USA). After the addition of 1 μL annexin V-FITC (Alexa Fluor^TM^ 488 conjugate, Invitrogen, Waltham, MA, USA) and 1 μL 7-ADD (Biolegend, San Diego, CA, USA), the cell mixture was incubated for 20 min at room temperature. A minimum of 10,000 cells per sample was collected on a BD Accuri^TM^ C6 flow cytometer (BD Biosciences, San Jose, CA, USA) and analyzed using the FlowJo Software v. 10.4.2 (TreeStar Inc., Ashland, OR, USA). Actinomycin D (80 µg/mL, Sigma) was used as a positive control for apoptosis.

Also, the activation of representative caspases of apoptosis pathways was evaluated by flow cytometry using the CaspGLOW^TM^ fluorescein active caspase-8 and 9 kits (Invitrogen, Waltham, MA, USA) according to the manufacturer’s instructions. For this, D-17 cells were plated in 24-well plates and synchronized for 24 h. Thereafter, the cells were treated with LEAS for 24 and 48 h, harvested by trypsinization, and stained with 1 μL Substrate-FITC (Caspase-Glo 8 or 9), incubated for 60 min at 37 °C in 5% CO_2_, and were finally washed twice with 100 μL of wash buffer. A minimum of 10,000 events per sample were collected on a BD Accuri^TM^ C6 flow cytometer (BD Biosciences, San Jose, CA, USA).

### 4.9. Cell Cycle Analysis

Cell cycle analysis was performed in D-17 cell line treated with LEAS using the BD cycle test Plus DNA kit (BD biosciences, San Jose, CA, USA) according to manufacturer’s specifications. Briefly, 5 × 10^4^ D-17 cells were seeded in 24-well plates and synchronized for 24 h. Further, the medium was replaced with 400 μL of fresh medium supplemented with heat-inactivated fetal bovine serum and treated with LEAS during 24 and 48 h. Then, cells were harvested following trypsinization and washed with buffer solution, adjusted to 1 × 10^5^ cells for treatment. Finally, each sample was centrifuged, and the supernatant was removed, solution A was added and incubated for 10 min at room temperature, then solution B was added and incubated for 10 min. Finally, solution C was added and incubated for 10 min in darkness and whilst being kept in ice-cold, until samples were analyzed on a BD Accuri^TM^ C6 flow cytometer (BD Biosciences, San Jose, CA, USA). For each sample, 20,000-gated events were acquired. Total event counts within the G0/G1, S, and G2/M phases were used to calculate the percentage of cells within each phase of the cell cycle.

### 4.10. Drug Combination Studies

The LEAS interactions with cytotoxic drugs usually used against canine osteosarcoma (cisplatin, carboplatin, or doxorubicin) were assessed. For this, D-17 cells were seeded (2500 cells/well) in a 96-well plate and then were subjected to the simultaneous treatment with LEAS (5, 10, 20, 50 µg/mL), cisplatin (3.34, 16.7, 33.4, 66.8 μM), carboplatin (50, 100, 200, 400 μM) or doxorubicin (46.8, 97.7, 187, 350 nM) and incubated for 72 h. The viable cells were determined by MTT assay as described above and the IC_50_ was estimated for each case. Controls consisting of cells treated with the individual compounds were included. All drugs were freshly prepared before each experiment. All experiments were performed in triplicates. Drug interactions were analyzed following the method of Aapro et al. [37]. The type of interaction between LEAS and cytostatic drugs was determined using the equations:

Additive = SF_l + y_ = SF_l_ × SF_y_

Sub-additive = SF_l_ × SF_y_ < SF_l + y_ < SF_l_ and SF_y_

Antagonistic = SF_l + y_ > SF_l_ × SF_y_

where, SF_l + y_ = surviving fraction of cells exposed to the combination of LEAS and cisplatin or carboplatin or doxorubicin, SF_l_ = surviving fraction of cells exposed to LEAS alone, SF_y_ = surviving fraction of cells exposed to cisplatin or carboplatin or doxorubicin alone. A combination index (CI) was determined and the CI values indicate a synergistic effect when <1, an antagonistic effect when >1, and an additive effect when equal to 1.

### 4.11. Statistical Analyses

The data were analyzed with the PRISM10.0 software, performing a one-way analysis of variance (One-Way ANOVA) using the post hoc Tukey test. The results are reported as the means ± the standard errors (SE), and the significance level was set at *p* ≤ 0.05.

## 5. Conclusions

LEAS was cytotoxic for the canine D-17 osteosarcoma cell line through the induction of caspase-dependent apoptosis. Also, LEAS increases the proportion of cells in G0/G1 phase. Besides, LEAS induced a significant loss of mitochondrial membrane potential and increased ROS production. LEAS improved the cytotoxicity of cisplatin, carboplatin, and doxorubicin on D-17 cells through a synergistic effect. These results show that avocado can be a source of attractive anticancer molecules and further studies are needed to evaluate its potential as an antitumor agent for bone cancer treatment in humans and dogs.

## Data Availability

The raw data supporting the conclusions of this article will be made available by the authors, without undue reservation, to any qualified researcher.

## Supplementary Materials

The following are available online, Figure S1: Effect of LEAS on MDCK cell line viability.

## Sample Availability

Samples of the compounds are not available from the authors.
