# Supplementary material for: Cytotoxicity of a Lipid-Rich Extract from Native Mexican Avocado Seed (Persea americana var. drymifolia) on Canine Osteosarcoma D-17 Cells and Synergistic Activity with Cytostatic Drugs
Source: Molecules. 2021 Jul 9;26(14):4178. doi: 10.3390/molecules26144178 (PMC8304388; doi:10.3390/molecules26144178)
Supplement: Supplementary file 1 [file molecules-26-04178-s001.zip › molecules-1285740-supplementary.pdf]

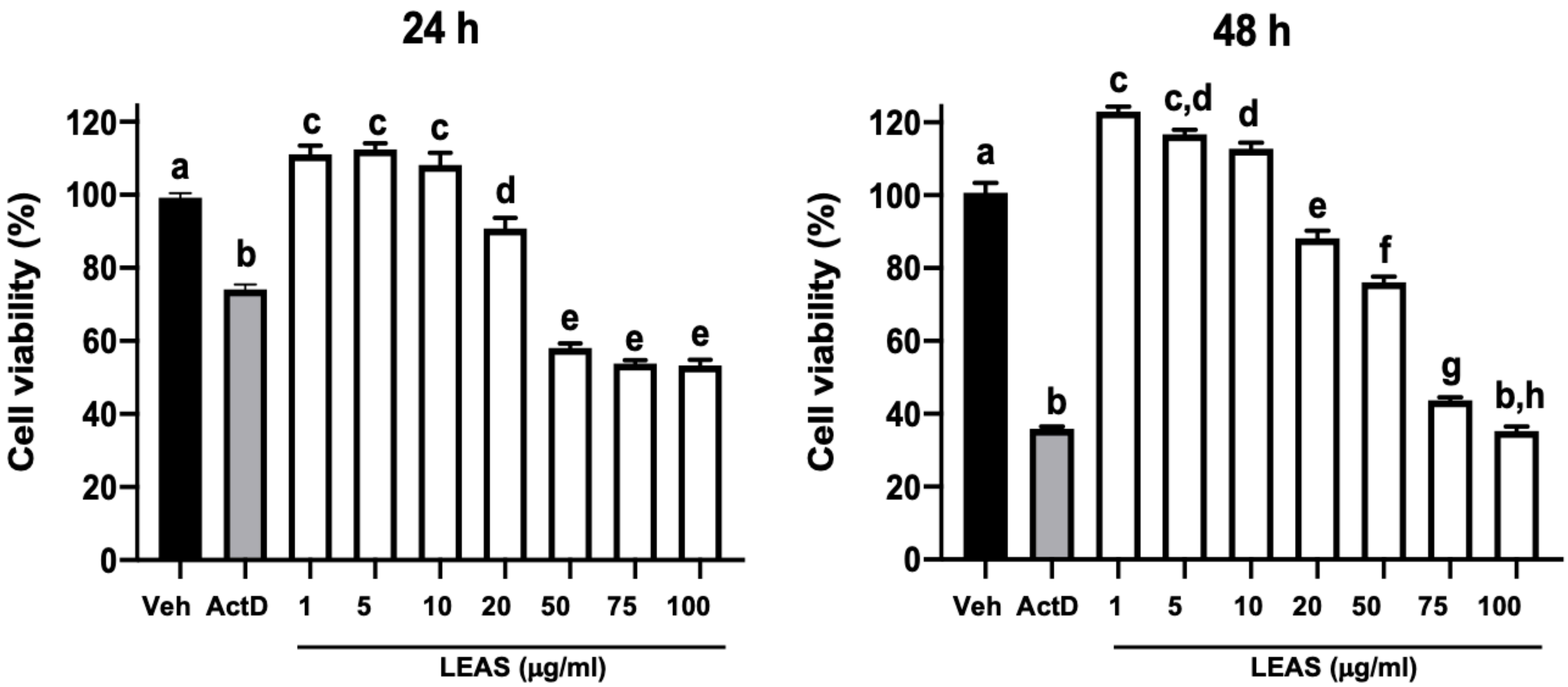

Figure S1. Effect of LEAS on MDCK cell line viability. Cells were treated with LEAS (1, 5, 10, 20, 50, 75, and 100 µg/ml) and cell viability was evaluated by MTT assays at 24 h and 48 h. Cell viability is shown with respect to cells treated with vehicle (DMSO 0.1%). Actinomycin D (ActD) was used as a positive control (80 µg/ml). Data represent the mean of three independent experiments performed in triplicate. Different letters denote significant differences within the treatments (one-way ANOVA and Tukey´s pairwise comparison, $P < 0.05$).
